# Experimental dataset on acid treated eggshell for removing cyanide ions from synthetic and industrial wastewaters

**DOI:** 10.1016/j.dib.2017.11.048

**Published:** 2017-11-17

**Authors:** Ghorban Asgari, Alireza Dayari

**Affiliations:** a Social Determinants of Health Research Center (SDHRC), Department of Environmental Health Engineering, Hamadan University of Medical Sciences, Hamadan, Iran; b Students Research Center, Hamadan University of Medical Sciences, Hamadan, Iran

**Keywords:** Eggshell membrane, Adsorption, Cyanide, Industrial wastewater, Synthetic wastewater

## Abstract

The data current in this article are associated to the efficacy of acid treated eggshell as eggshell membrane (ESM) as an adsorbent for eliminating cyanide ion from synthetic and industrial wastewaters. This article describes the effects of selected factors such as pH (3–11), contact time (5–60 min), cyanide ion concentrations (50–150 mg/L), ESM dose (0.25–2 g/L), and solution temperature (20–50) on the removal cyanide ion from aqueous solution. The maximum cyanide ion removal obtained at a solution pH of 9–11. The kinetic data agreed with the pseudo-second-order kinetic. The equilibrium adsorption data at different temperatures well set through Langmuir equation. FTIR and thermodynamic data describe main adsorption phenomenon in cyanide ion onto ESM could be the ion exchange and chemisorption.

**Specifications Table**

**Table:** 

Subject area	Environmental engineering
More specific subject area	Environmental technology and waste management
Type of data	Table, image, and figure
How data was acquired	The capability of eggshell membrane to adsorb cyanide ions was conducted using a series of batch tests in a shaker- incubator instrument.
Data format	Analysis
Experimental factors	Monitoring cyanide ions concentrations under various levels of initial target concentration, pH, adsorbent mass temperature, and reaction time for achieving the optimal conditions to remove cyanide ion from wastewater using eggshell membrane.
Experimental features	Cyanide ion adsorption by eggshell membrane and introduce low -cost and applied waste material in wastewater treatment
Data source location	Chemistry laboratory water and wastewater, Hamadan University of Medical Sciences, Hamadan, Iran.
Data accessibility	Data are accessible in the article

**Value of the data**

• The data presents a low -cost adsorbent make from waste material of eggshell membrane.
• The isotherm, kinetic and thermodynamic data will be useful and valuable for expecting and modeling the adsorption capacity and mechanism of cyanide ion elimination via the adsorbent. The attained data will be beneficial for the methodical and engineering community that needing to scale up and design an adsorption column with eggshell membrane as bed for the elimination of cyanide ion from water or wastewater.

## 1 Data

**Fig. 1:**
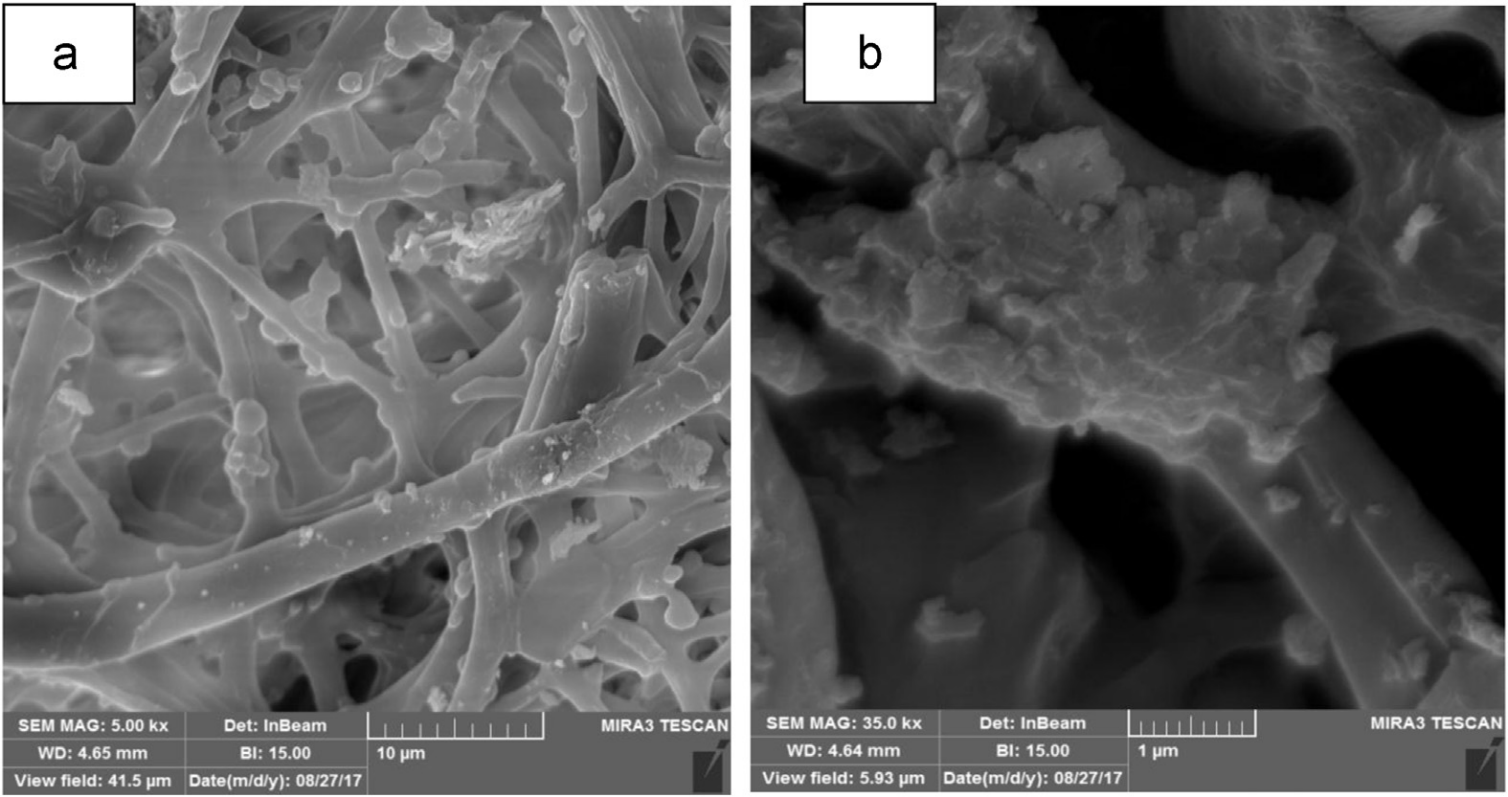
SEM analysis data for make eggshell membrane (a) 10 µm magnification, (b) 1 µm magnification.

**Fig. 2:**
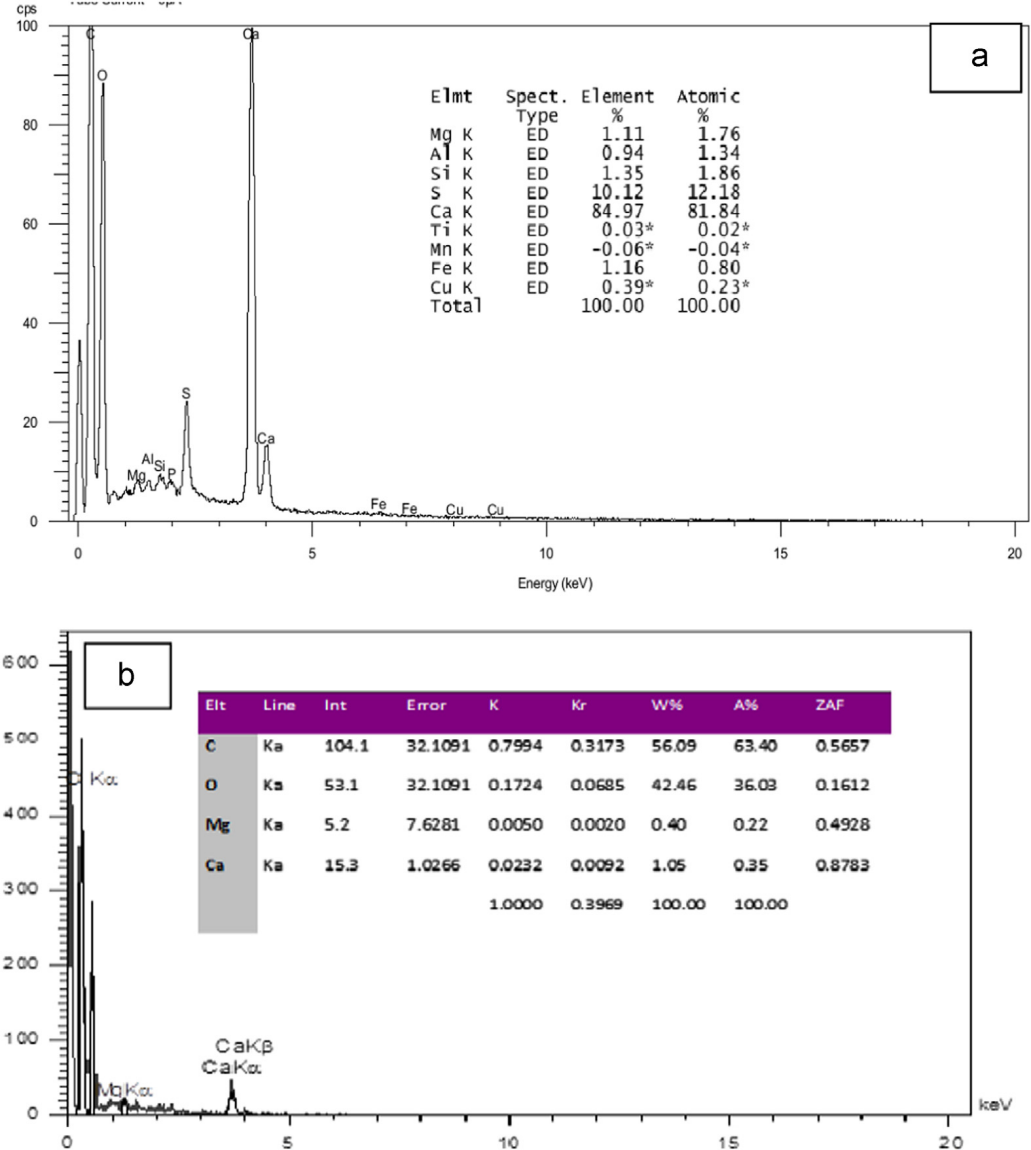
EDX spectra of the fresh eggshells (a) and (b) ESM.

**Fig. 3:**
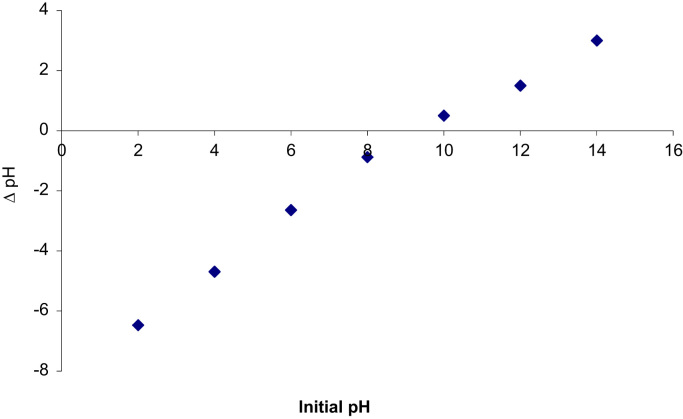
Experimental data for point of zero charge (pHzpc) of ESM.

**Fig. 4:**
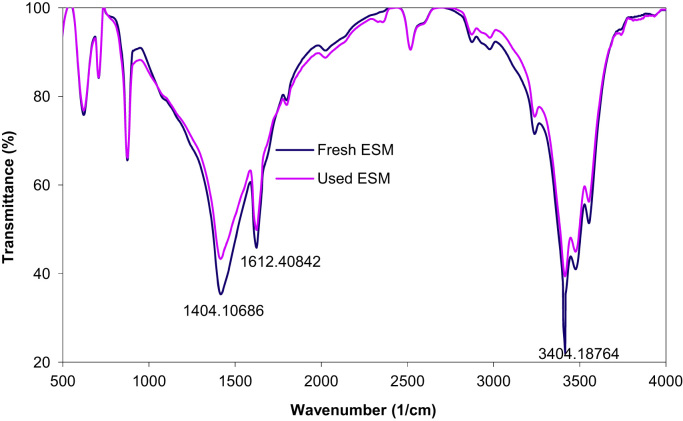
The FTIR spectrum of ESM before and after cyanide ion adsorption.

**Fig. 5:**
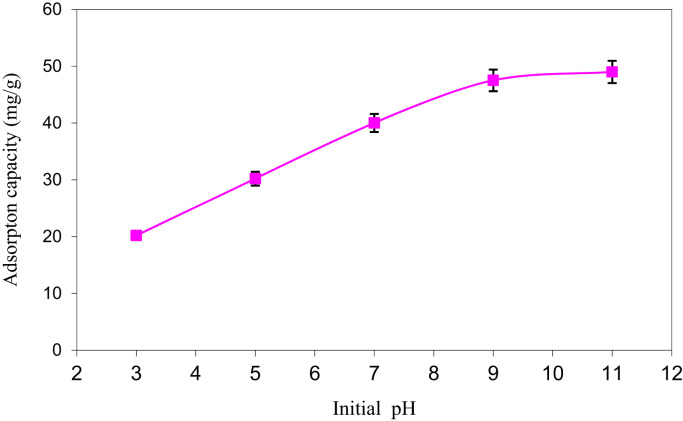
The influence of solution pH on cyanide removal by ESM.

**Fig. 6:**
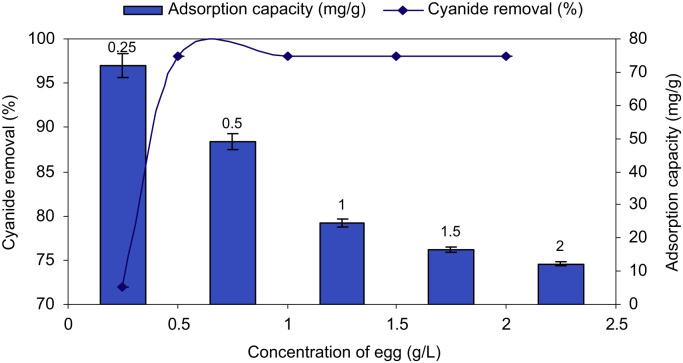
The profile of cyanide removal as a function of ESM dose (cyanide concentration=100 mg/L, solution pH=11).

**Fig. 7:**
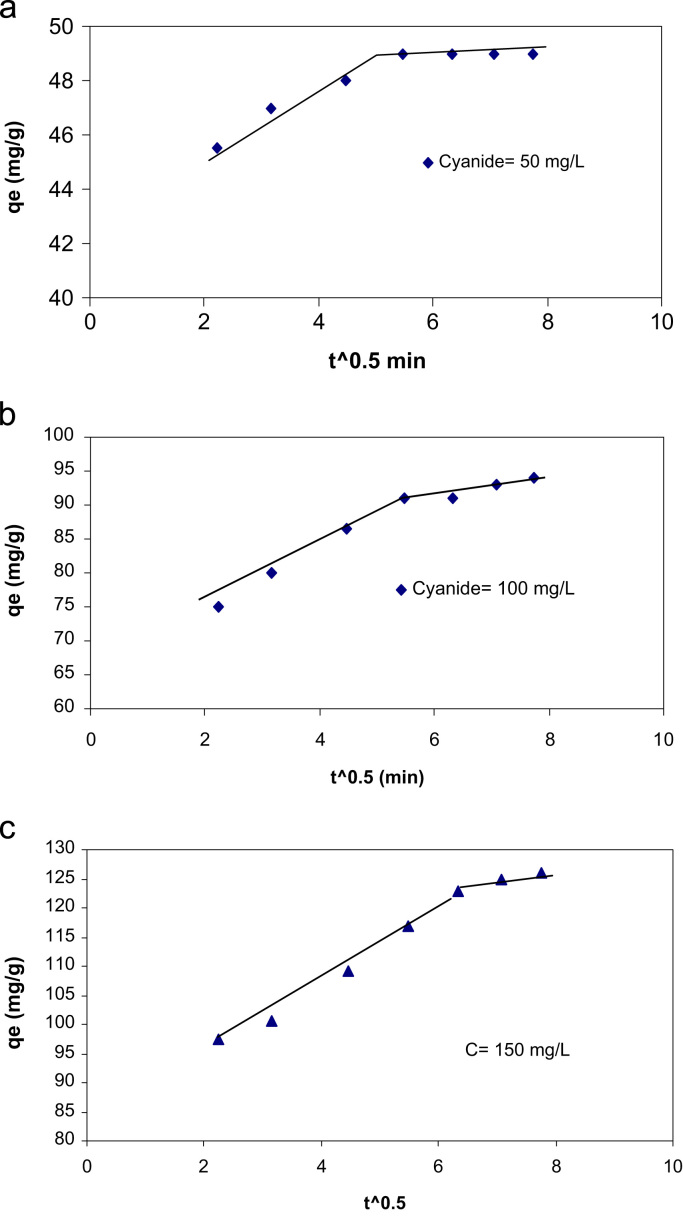
Intraparticle diffusion model plot for the adsorption of cyanide onto ESM by different concentrations (a) 50 mg/L, (b) 100 mg/L, and (c) 150 mg/L of cyanide.

**Fig. 8:**
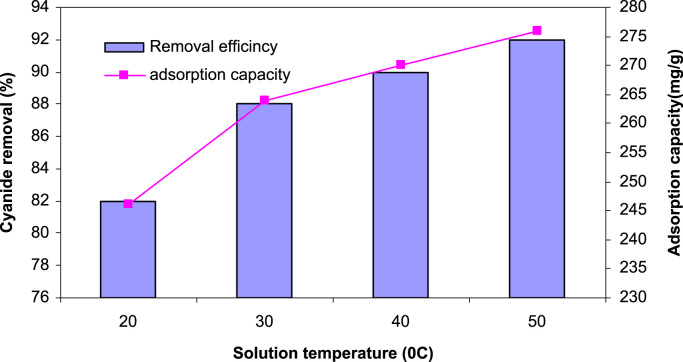
The profile of cyanide ion removal as a function of solution temperature.

SEM data for eggshell membrane with different magnification were shown in Fig. 1a, b. Fig. 2a, b displayed EDX spectra data of the fresh eggshells and ESM. Fig. 3 indicated experimental data for point of zero charge (pHzpc) of ESM. Fig. 4 depicted data for the FTIR spectrum of ESM before and after cyanide loaded. Data of the influence of solution pH on cyanide ion removal by ESM is shown in Fig. 5. In Fig. 6 the profile of cyanide removal data as a function of ESM dosage indicated. Fig. 7 demonstrated data of intraparticle diffusion model plot for the adsorption of cyanide onto ESM through different concentrations. Fig. 8 showed the profile of cyanide ion removal data as a function of solution temperature. The parameters obtained from pseudo- first -order model parameters with different initial cyanide ion concentrations are tabulated in Table 1. The parameters obtained from pseudo-second-order model parameters with different initial cyanide ion concentrations indicated in Table 2. The parameters obtained from intraparticle diffusion model with different initial cyanide ion concentrations exhibited in Table 3. In Table 4 the data regarding to Langmuir, Freundlich, Dubinin–Radushkevich and Temkin adsorption isotherm parameters are presented. Thermodynamic data for adsorption of cyanide ion on ESM indicated in Table 5. Table 6 described the quality of electroplating plant wastewater before and after treatment with the ESM.

**Table 1:** The parameters obtained from pseudo- first -order model parameters with different initial cyanide ion concentrations.

Concentration (mg/L)	*q*_e_,_meas_ (mg/g)	*q*_e_,_calc_ (mg/g)	*k*_1_	ARE	APE	SSE	*R*^2^	Rate equation (Fitted model)
50	49	3.3	0.072	30.5	46.67	6.77	0.865	In(qe,meas-qt)=1.939−0.072t
100	94	35	0.056	40.55	60.62	8.79	0.938	In(qe,meas-qt)=3.555−0.056t
150	126.6	51	0.064	55.8	80.60	10.51	0.978	In(qe,meas-qt)=3.932−0.064t

**Table 2:** The parameters obtained from pseudo-second-order model parameters with different initial cyanide ion concentrations.

Concentration (mg/L)	*q*_e_,_meas_ (mg/g)	*q*_e_,_calc_ (mg/g)	*k*_2_	ARE	APE	SSE	*R*^2^	Rate equation (Fitted model)
50	49	49.5	0.044	5.77	2.33	.078	0.999	*t*/*q*_t_=0.009275+0.020t
100	94	96.3	0.007	8.55	3.33	0.08	0.999	*t*/*q*_t_=0.015405+0.010t
150	126.6	131	0.003	8.67	4.44	0.09	0.998	*t*/*q*_t_=0.019424+0.008t

**Table 3:** The parameters obtained from intraparticle diffusion model with different initial cyanide ion concentrations.

Concentration (mg/L)	*k*_id_	*R*^2^	Rate equation (Fitted model)
50	0.616	0.867	qt=0.616t0.5+44.48
100	3.426	0.936	qt=3.426 t0.5+69.34
150	5.706	0.987	qt=5.708 t0.5+85.91

**Table 4:** Langmuir, Freundlich Dubinin–Radushkevich and Temkin adsorption isotherm parameters.

Isotherm model	Parameters	Equilibrium temperature (°C)			
		20	30	40	50
Langmuir	*K*_L_	0.0058	0.048	0.004	0.0043
	*q*_max_	166.25	169.94	188.67	294.12
	RL	0.077	0.41	0.45	0.45
	*R*^2^	0.998	0.988	0.987	0.985
	RMSE	5.70	6.05	6.99	7.88
	*X*^2^	0.26	0.55	0.78	0.89
Freundlich	*K*_F_	80.35	48.55	45.65	40.23
	1/n	0.28	0.31	0.36	0.40
	*R*^2^	0.956	0.945	0.954	0.927
	RMSE	24.35	27.67	29.89	30.55
	*X*^2^	28.24	34.26	38.85	39.01
Dubinin–Radushkevich	*K*_DR_	0.002	0.004	0.008	0.007
	*E*	15	11	8	8.5
	*R*^2^	0.856	0.866	0.883	0.843
	RMSE	29.63	35.81	36.73	38.67
	*X*^2^	42.48	45.70	46.8	50.12
Temkin	BT	8	12	15	25
	AT	3.23	3.64	12.6	14.32
	*R*^2^	0.942	0.953	0.952	0.952
	RMSE	16.45	18.17	20.78	23.34
	*X*^2^	5.55	8.91	12.67	14.77

**Table 5:** Thermodynamic data for adsorption of cyanide ion on ESM.

Cyanide concentration (mg/L)	Δ*H*° (kJ/mol)	Δ*S*° (kJ/(mol K))	Δ*G*° (kJ/mol)			
			293	303	313	323
50	31.45	80	−55.6	−56.3	−57.6	−58.3
100	40.34	132	−59.4	−61.1	−62.4	−63.5
150	73.766	148	−64.2	−66.7	−67.8	−68.1

**Table 6:** The quality of electroplating plant wastewater before and after treatment with the ESM (ESM amount: 0.5 g/L and contact time: 60 min).

Parameters	Unit	Value	
		Raw wastewater	Treated wastewater
Cyanide	mg/L	76	<0.2
BOD_5_	mg/L	151	45.1
pH	–	7.8	7.7
Turbidity	NTU	6	4
Nitrate	mg/L	12	10
Chromate	mg/L	19	3
Sulfate	mg/L	183	179
Salinity	%	0.96	0.91

## 2 Experimental design, materials and methods

### 2.1 Materials

In this work, fresh eggshells used were obtained from local confectionary shop. The eggshells were initially washed with tap water and then dried at 105 °C. The dried eggshells were grinded to size 0.5–0.6 mm. To prepare ESM, the eggshells were occupied in the hydrochloric acid (%0.5) for 35 min [1]. The obtained ESM were washed with distilled water and washed ESM was dried in the oven at 80 °C. The stock of cyanide solution (1.0 g CN^−^/L) was prepared by dissolving required quantity of NaCN in 1.0 L of Milli-Q water. All of chemicals and reagents were of analytical grade that were used without further purification (Merck Co., Germany).

### 2.2 Adsorption tests

The ability of ESM to the cyanide removal was assessed by a series of batch experiments in a shaker- incubator instrument (Pars Azma Co, Iran). For each experimental run, 100 mL of solution having a known concentration of cyanide ion and with the chosen level of pH was first poured into beaker. Then, a fixed mass of ESM was added to vessel and placed inside the shaker-incubator. Next, vessel was mixed at 120 rpm for a given time. Lastly, the suspension of shacked sample was filtrated and analyzed for the concentration remained cyanide ion. The influence of temperature, pH, mixing time, initial cyanide ion concentration and adsorbent mass as variable parameters assessed. Eqs. (1), (2) were used to determine the cyanide removal efficiency (R) and the adsorption capacity of ESM in each run [2], [3].

(1) $$R(\%)=\left(\frac{C_{0}-C_{e}}{C_{0}}\right)\times 100$$

(2) $$q_{e}(mg/g_{ESM})=\frac{V}{M}\times (C_{0}-C_{e})$$

where *C*_0_ and *C*_e_ are the initial and equilibrium concentration of cyanide ion, respectively; *q*_e_ is equilibrium cyanide concentration on ESM, *V* is the volume of solution and *M* is the mass of the used ESM sample.

### 2.3 Analysis and characterization

Chemical composition ESM was investigated using a Philips model XL-30 scanning electron microscope (SEM) with energy-dispersive X-ray microanalysis (EDX). The pH of point of zero charge (pHpzc) for ESM was measured by the method described by Asgari et al. [4]. Fourier transform infrared (FTIR) spectroscopy (Perkin–Elmer spectrophotometer spectrumone) in the range, 450–4000 cm^-1^ was used to investigate of functional groups on the surface of ESM. The concentration of cyanide ion was determined according to standard method 4500-CN-D of APHA [5].

### 2.4 Isotherm of cyanide ion adsorption onto ESM

To describe the cyanide adsorption capacity data, obtained isotherm data were fitted by four most commonly used isotherms including Langmuir, Freundlich, Dubinin-Radushkuvich and Temkin. The linear forms of apply isotherms equations can be represented respectively as bellow [5]:

(3) $$\mathrm{Freundlich}\;\text{equation}:\;\log\;q_{e}=\;\log\;K+\frac{1}{n}\;\log\;C_{e}$$

(4) $$\mathrm{Langmuir}\;\mathrm{equation}:\;\frac{1}{q_{e}}=\frac{1}{q_{\max}\times bC_{e}}+\frac{1}{q_{\max}}$$

(5) $$\mathrm{Temkin}\;\mathrm{equation}:\;q_{e}=BLnK_{t}+BLnC_{e}$$

(6) $$\text{Dubinin}–\text{Radushkevich}\;\mathrm{equation}:\;Lnq_{e}=Lnq_{\max}-k\varepsilon^{2}$$

where *q*_e_ and *C*_e_ are parameters that are described in Eqs. (1), (2). K and n are constants that indicate the adsorption capacity and the adsorption intensity. *q*_max_ is the maximum amount of adsorption (mg/g) and b is the adsorption equilibrium constant (L/mg). $B=\frac{RT}{b}$, *T* is the absolute temperature in *K* and *R* is the universal gas constant in (J/mol K). *Ɛ* (Polanyi potential) is RT ln(1+(1/C_e_), *q*_max_ the adsorption capacity (mg/g), *k* a constant related to adsorption energy, *R* and *T* are the gas constant and temperature (K). *R*_L_ equilibrium constant obtained as follows [5], [6], [7]:

(7) $$R_{L}=\frac{1}{1+bC_{0}}$$

where *C*_0_ is the initial concentration of cyanide ion.

*k* as energy adsorption, calculated from the *k* value using the following equation:

(8) $$E=\frac{1}{\sqrt{2k}}$$

### 2.5 The kinetic study

To investigate the adsorption mechanism of cyanide removal, the experimental data was fitted with most commonly used pseudo-first-and second-order kinetics model at different experimental conditions. The pseudo first-order kinetic linear equation is generally as follow:

(9) $$In(q_{e,meas}-q_{t})=In(q_{e,calc})-k_{1}t$$

where $q_{e,meas}$ and $q_{t}$ are experimentally measured and calculated cyanide adsorbed on ESM at time *t*, *k*_1_ is the rate constant for pseudo-first-order kinetic. The linear regression analysis of In (*q*_e,meas_−*q*_t_) vs t for different experimental conditions will give the data of the *q*_e,calc_ (*q*_e,calc_=exp (intercept)) and *k*_1_ (*k*_1_=−(slop)) (8), (9).

The pseudo second-order kinetic linear equation is generally as follow:

(10) $$\frac{t}{q_{t}}=\frac{1}{k_{2}q_{e,calc}^{2}}+\frac{1}{q_{e,calc}}t$$

The value of *q*_e,calc_ (*q*_e_,_calc_=1/slope) and *k*_2_ as the rate constant (*k*_2_=slope^2^/intercept) of the pseudo-second-order equation obtained from linear regression analysis of *t*/*q*_t_ *vs* t [8], [9], [10]. Via Weber and Morris equation was also used to evaluate experimental adsorption kinetic data. The linear form of the equation is as follows [11], [12]:

(11) $$q_{t}=k_{id}t^{0.5}+C$$

where *C* is the intercept and kid is the intraparticle rate constant obtained from the slope of the plot of *q*_t_ against *t*^0.5^.

### 2.6 Thermodynamics study

To explain the mechanism of cyanide adsorption onto eggshells, the thermodynamics parameters associated with the adsorption were determined by using following equation [13]:

(12) $$\Delta G^{\circ }=-\;RT\;\ln\;K_{\circ }$$

(13) $$K_{\circ }=\frac{q_{e}}{Ce}$$

(14) $$\ln\;K_{\circ }=-\frac{\Delta H^{\circ }}{RT}+\frac{\Delta S^{\circ }}{R}$$

### 2.7 Validity of adsorption isotherm and kinetic study

The applicability of the isotherm equations and kinetic models were evaluated by The correlation coefficient and also comparing residual root mean square error (RMSE) and the chi-square test (*X*^2^) (3, 15) which can be described as:

(15) $$RMSE=\sqrt{\frac{1}{n-2}{\sum_{i=1}^{N}\left(q_{e,meas}-q_{e,calc}\right)}^{2}}$$

(16) $$\chi^{2}=\sum_{i=1}^{N}\frac{{\left(q_{e,meas}-q_{e,calc}\right)}^{2}}{q_{e,calc}}$$

The correlation coefficient (*R*^2^) and also the average relative error (ARE), the sum of squares error (SSE) and the average percentage error (APE) in the kinetics studies use to the validity of kinetic models data and they were calculating by following Eqs:

(17) $$ARE={\sum_{i=1}^{N}\left[\frac{q_{e,meas}-q_{e,calc}}{q_{e,meas}}\right]}_{i}$$

(18) SSE=∑i=1N(q−e,calcqe,meas)

(19) $$APE=\sum_{i=1}^{N}\frac{\left[\left(q_{e,meas}-q_{e,calc}\right)/q_{e,meas}\right]}{N}i\times 100$$

where $q_{e,meas}$ is the observation from the batch experiment *i* and *N* is the number of measurements made. Upon completion of the basic adsorption experiments, data of the efficacy of ESM in the removal of cyanide ion from industrial wastewater was evaluated. For this, a bulk wastewater sample was obtained from a local electroplating plant.

## Figures and Tables

**Figure:**
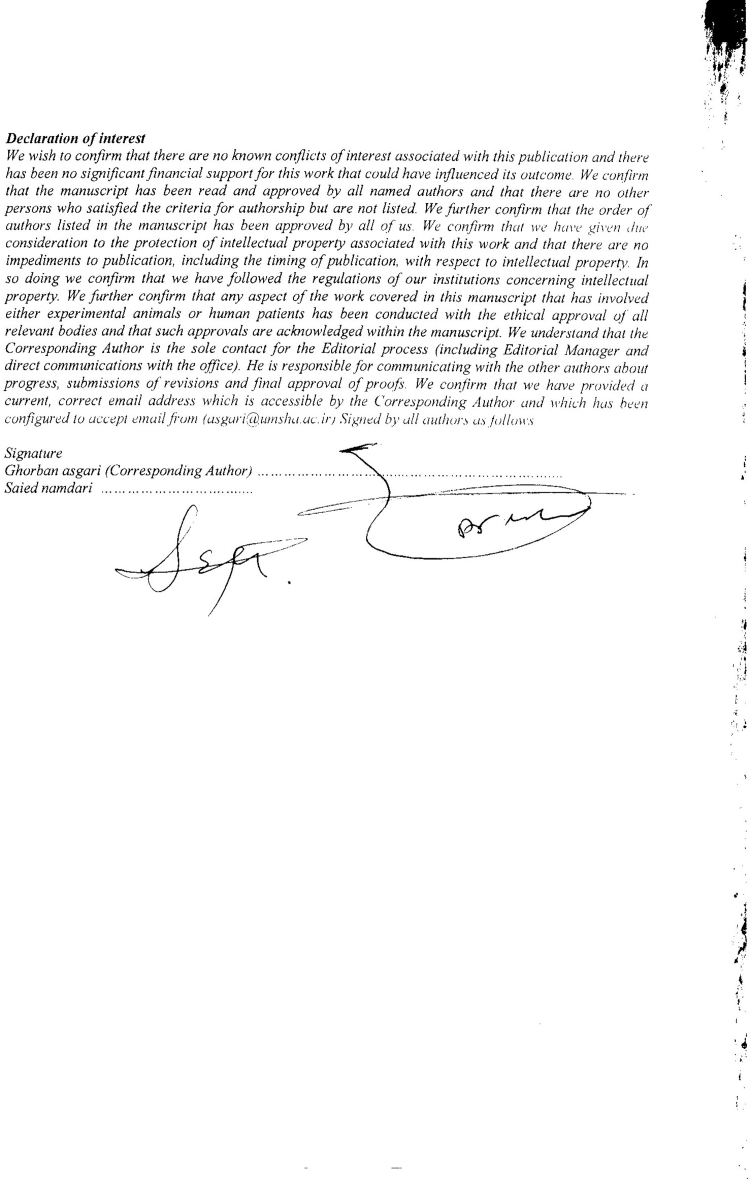
Supplementary Figure.
